# The Duodenum Under Duress: Unmasking Isolated Duodenal Hypoperfusion After Kidney Transplantation

**DOI:** 10.7759/cureus.98019

**Published:** 2025-11-28

**Authors:** Druthisree Veldi, Vibhu V Mittal, Devamsh Govinda Narayanareddy, Varun Verma

**Affiliations:** 1 Gastroenterology, Max Super Speciality Hospital, New Delhi, IND; 2 Nephrology, Max Super Speciality Hospital, New Delhi, IND

**Keywords:** acute gi bleed, duodenal ischemia, non-occlusive mesenteric ischemia, post kidney transplant, type 1 diabetes mellitus (t1d)

## Abstract

Duodenal ischemia is an uncommon clinical entity due to the extensive collateral blood supply of the duodenum. It is rarely reported in the postoperative setting and poses a significant diagnostic and management challenge. We present the case of a 34-year-old male patient with uncontrolled type 1 diabetes mellitus and end-stage renal disease on maintenance hemodialysis, who underwent a robot-assisted living donor kidney transplant. Postoperatively, he developed cardiogenic shock, requiring triple inotropic support, mechanical ventilation, and anticoagulation. During recovery, he developed melena with a significant drop in hemoglobin. Upper gastrointestinal endoscopy revealed circumferential duodenal ulceration with necrotic mucosa, raising suspicion of duodenal ischemia. CT angiography showed reduced mucosal enhancement in the duodenum and proximal jejunum, without evidence of major vascular occlusion. Non-occlusive mesenteric ischemia was considered the presumed mechanism for duodenal ischemia. The patient was initially managed conservatively; however, due to persistent gastrointestinal dysfunction, diagnostic laparotomy and feeding jejunostomy were performed. Intraoperative findings confirmed ischemic changes in the duodenum with a clear demarcation at the duodenojejunal flexure. Despite all supportive measures, the patient succumbed to sepsis a week later. This case highlights the rare occurrence of duodenal ischemia in a young postoperative patient and underscores the importance of early recognition and multidisciplinary management in improving outcomes.

## Introduction

We would like to present a rare case of postoperative duodenal ischemia, a condition infrequently encountered in clinical practice due to the duodenum’s extensive collateral vascular supply. Acute mesenteric ischemia (AMI) is characterized by a sudden interruption of blood flow to portions of the bowel, resulting in mucosal injury, inflammation, and, if not promptly addressed, transmural necrosis [1]. While ischemia can affect any segment of the gastrointestinal tract, duodenal involvement is exceedingly rare, constituting only a small fraction of these cases. The duodenum is particularly well protected from ischemia due to its dual arterial supply from the gastroduodenal artery (a branch of the celiac trunk) and the inferior pancreaticoduodenal artery (from the superior mesenteric artery). This robust collateralization typically compensates even in settings of reduced systemic perfusion or partial vessel occlusion. As a result, documented cases of duodenal ischemia, especially in the postoperative period, are extremely scarce in the literature [2].

Due to its rarity, duodenal ischemia lacks standardized diagnostic and management protocols. The presentation is often non-specific, including upper abdominal pain, gastrointestinal bleeding, or signs of obstruction or perforation, making clinical suspicion essential. Imaging modalities such as contrast-enhanced CT scans and endoscopy may aid in diagnosis, although findings can be subtle. Management varies from conservative treatment to surgical intervention, depending on the extent of injury and the underlying etiology [2].

We report this case to contribute to the limited literature on duodenal ischemia in the postoperative setting and to highlight the importance of early recognition and individualized management in such unusual presentations.

## Case presentation

**Figure 1:**
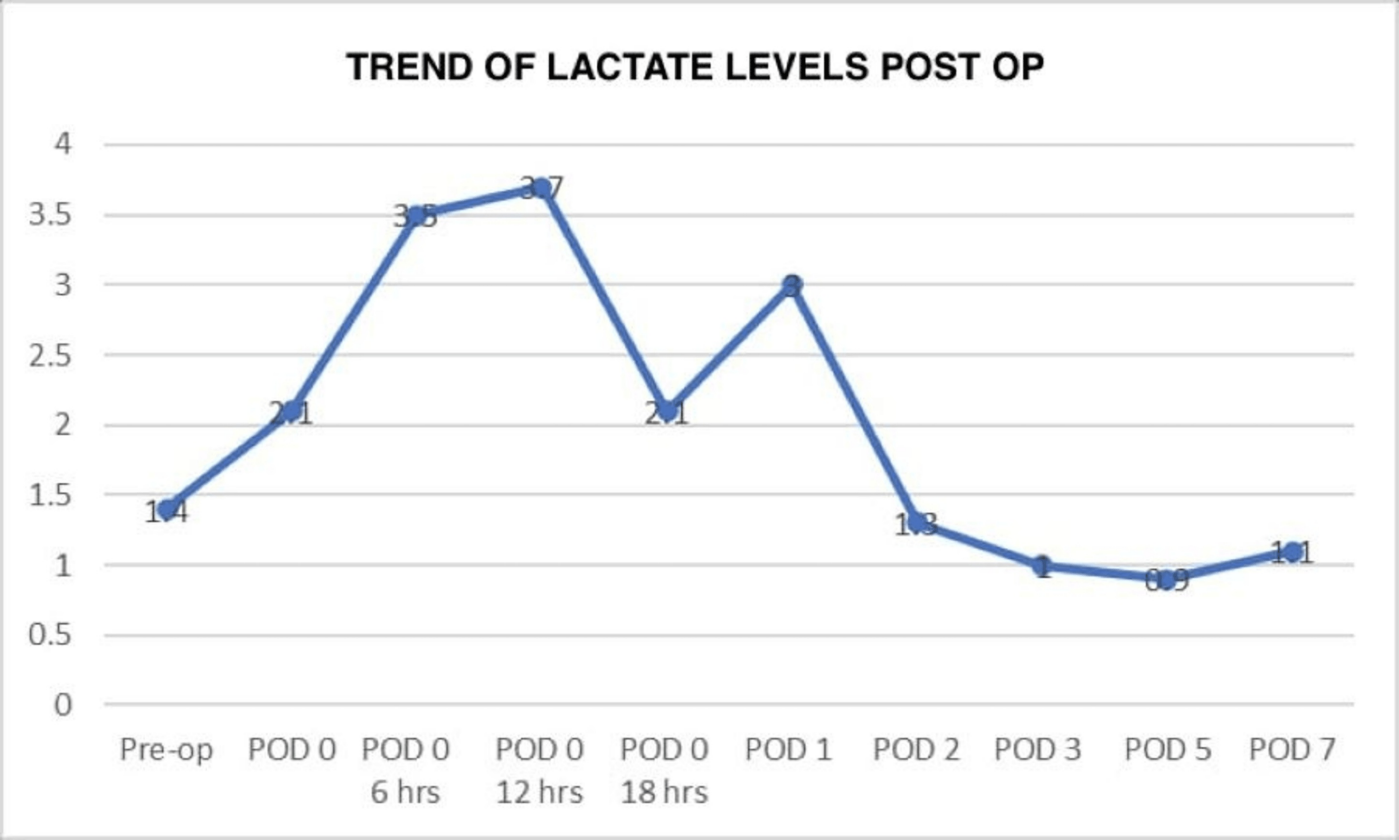
Trend of lactate levels

A 34-year-old man, a known case of poorly controlled type 1 diabetes mellitus (HbA1c 9.6) with advanced microvascular complications, diabetic retinopathy, diabetic neuropathy, diabetic foot post right great toe amputation, and end-stage diabetic nephropathy on maintenance hemodialysis, was evaluated for a living-related donor kidney transplant. Following pre-anesthesia clearance, he underwent robot-assisted kidney transplant surgery. In the immediate postoperative period, the patient’s course was stormy. On postoperative day one, his urine output remained suboptimal, and he developed hemodynamic instability, requiring escalation to triple inotropic support (noradrenaline, adrenaline, and dobutamine). On evaluation, he was found to have acute coronary syndrome, with elevated troponin I, creatine kinase-MB, and lactate levels (see Figure 1), and echocardiography showing a left ventricular ejection fraction of 15-20%.

**Figure 2:**
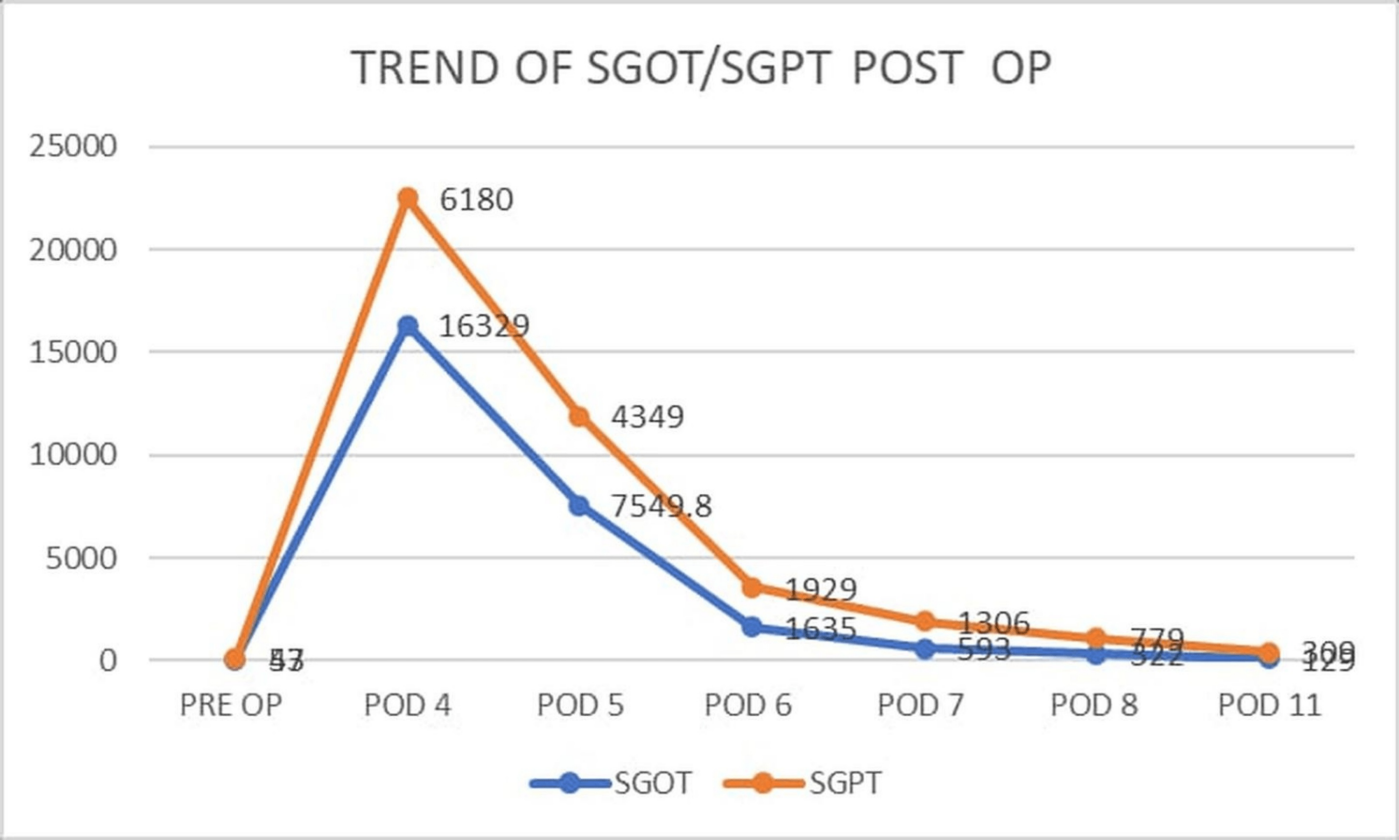
Trend of liver enzymes SGOT: Serum glutamic-oxaloacetic transaminase; SGPT: serum glutamic pyruvate transaminase

He was intubated, mechanically ventilated, and started on anticoagulation (dalteparin sodium 2500IU twice a day subcutaneously) and intensive supportive care. In view of deranged liver function tests (see Figure 2), a gastroenterology consultation was sought, and the patient was diagnosed with ischemic hepatitis.

Over the next three days, his cardiac function improved, and vasopressors were gradually weaned off. However, he remained ventilator-dependent.

**Figure 3:**
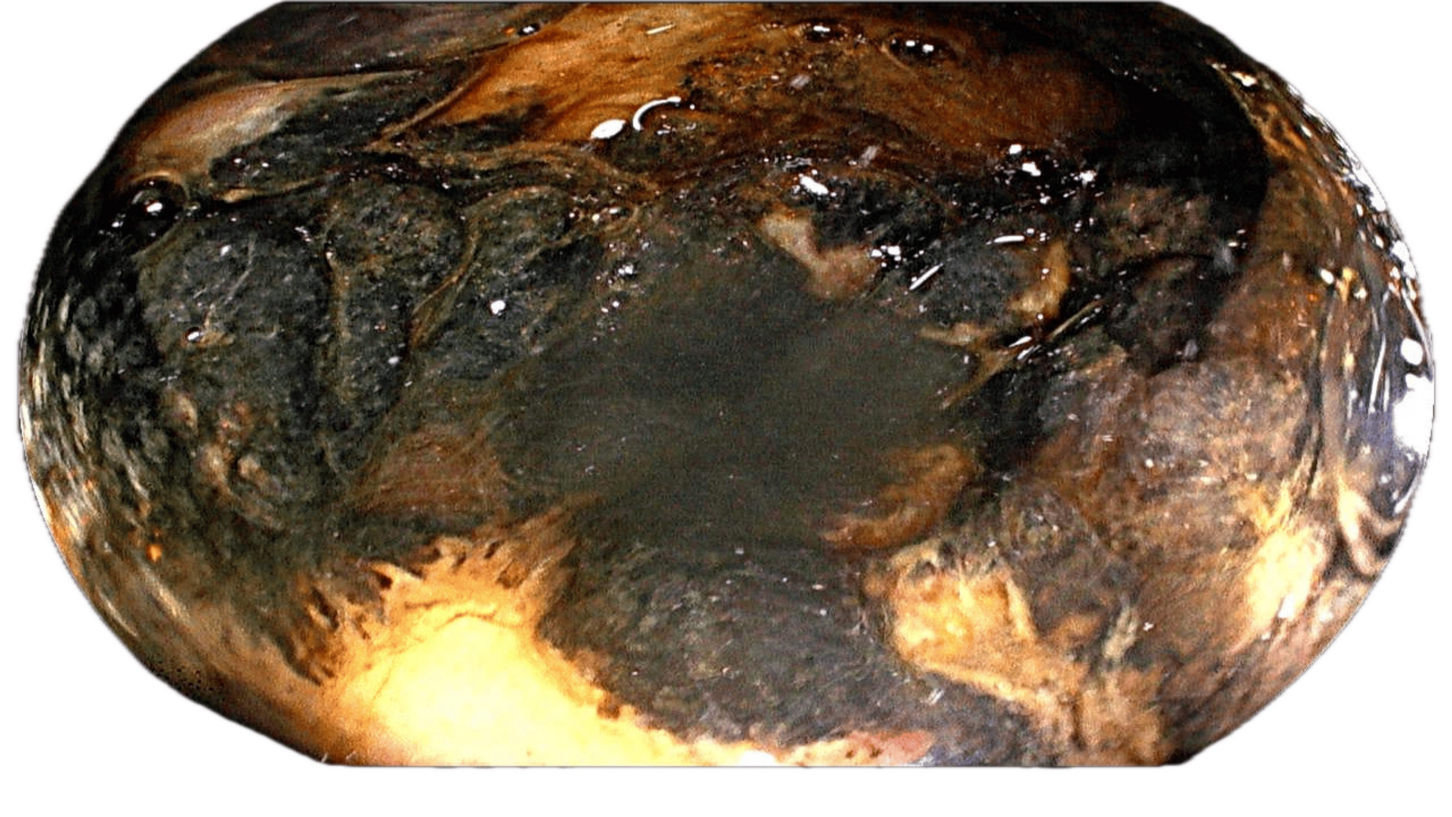
Endoscopic image of the duodenal ischemia

Amidst slow clinical recovery, on postoperative day four, a new complication emerged, melena with a significant drop in hemoglobin. Upper gastrointestinal endoscopy revealed linear mucosal breaks in the distal esophagus, a normal stomach, and extensive circumferential ulceration with necrotic, friable mucosa in the duodenal bulb and D1-D2 duodenal junction (see Figure 3).

Although no active bleeding was seen, the findings raised a strong suspicion of duodenal ischemia.

**Figure 4:**
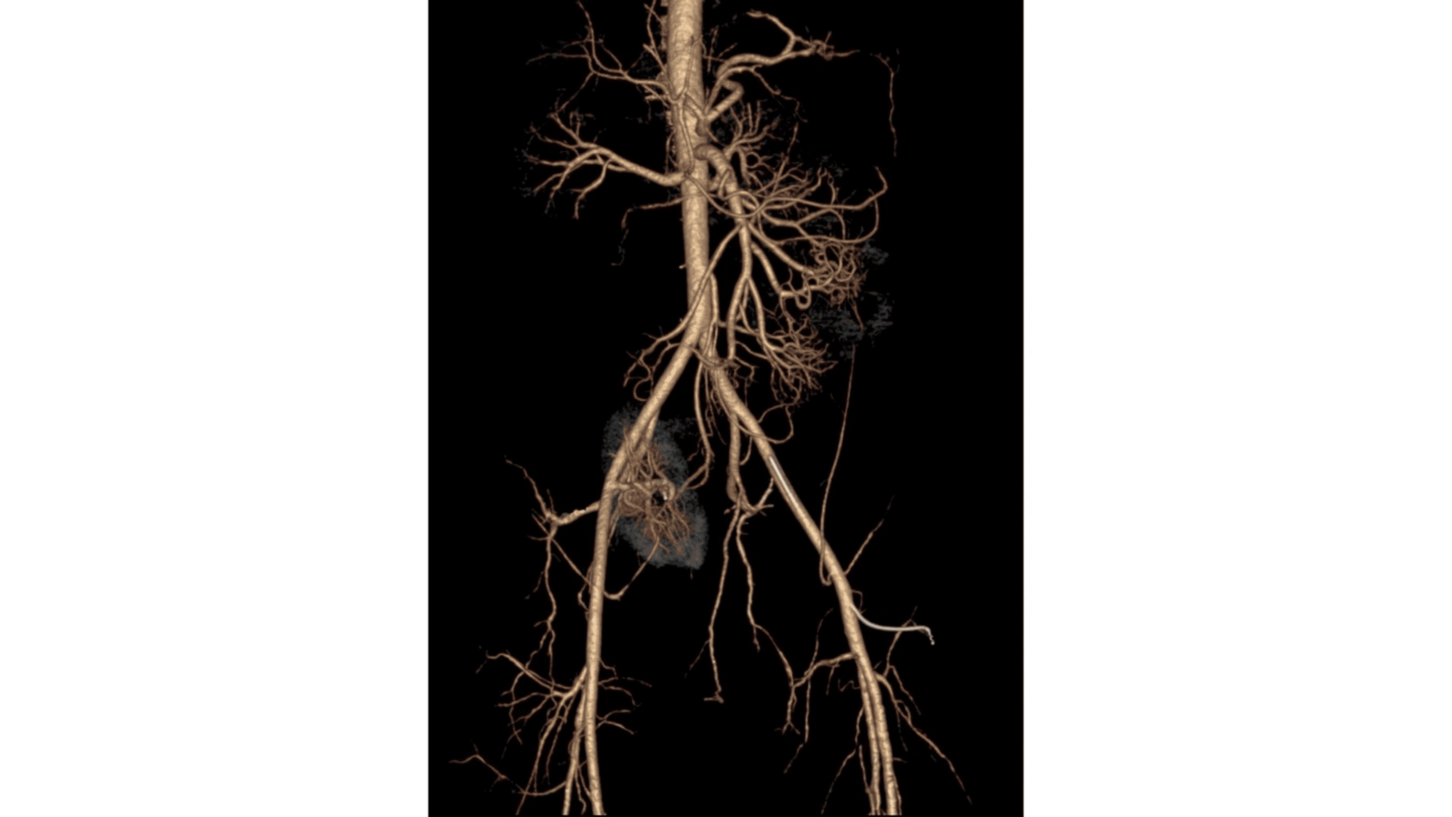
CT angiography image of the abdominal arteries

A CT angiography was performed, which ruled out major vascular occlusion but revealed reduced mucosal enhancement in the duodenum, duodenojejunal (DJ) flexure, and a proximal jejunal loop, findings suggestive of segmental ischemia (see Figure 4).

**Figure 5:**
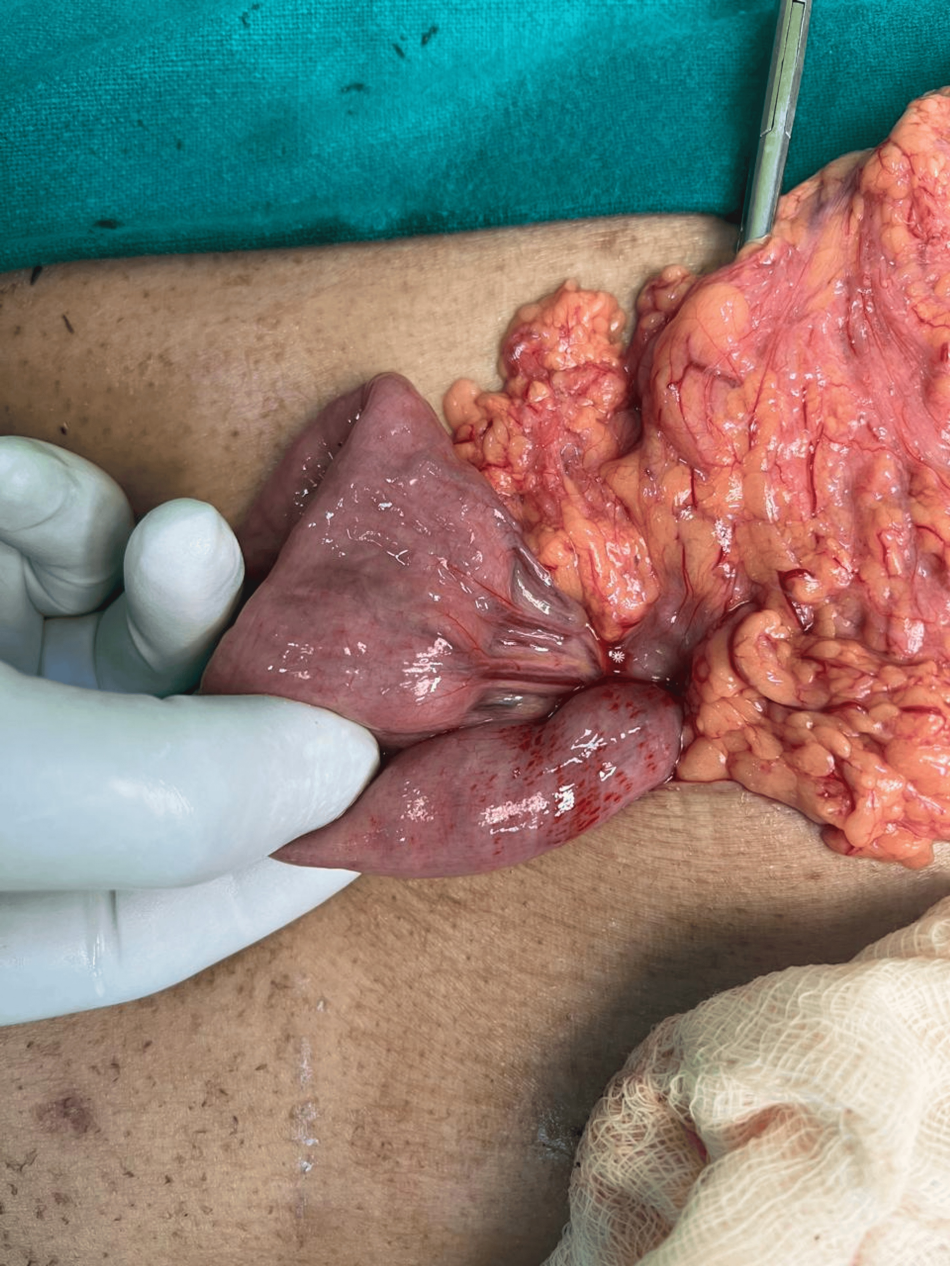
Intraoperative image of the duodenojejunal flexure

The patient was managed conservatively with proton pump inhibitor infusion, bowel rest, IV antibiotics, and supportive care. However, given the risk of perforation and lack of clinical improvement, a multidisciplinary team decision was made to proceed with diagnostic laparotomy. The patient underwent midline laparotomy. Intraoperative findings revealed ascites, dusky discoloration of the duodenum, and a clear ischemic demarcation at the DJ flexure (see Figure 5).

There was improvement in color after rewarming the bowel with warm saline. The surgeon decided against resection of the duodenum. A feeding jejunostomy was successfully performed. Despite comprehensive medical and surgical management, the patient’s condition deteriorated due to fungal septicemia with septic shock (blood culture growing Candida species), and he succumbed a week later. This case illustrates the rare occurrence of duodenal ischemia in the postoperative period, even in the absence of large vessel occlusion, and underscores the complexity of managing such cases in critically ill patients with multiple co-morbidities.

## Discussion

While the most common cause of chronic mesenteric ischemia impacting the celiac axis or superior mesenteric artery is atherosclerotic disease, AMI is most commonly the result of superior mesenteric artery embolization, superior mesenteric artery thrombosis, non-occlusive mesenteric ischemia (NOMI), or acute mesenteric venous thrombosis, which does not typically affect the duodenum [1]. A review of the literature reveals that duodenal ischemia is a rare condition, with most reported cases occurring in elderly patients with significant co-morbidities or following major vascular or spinal surgeries [3-5]. Gupta et al. described a case of diffuse duodenal ischemia following spinal surgery in an elderly woman with postoperative hypotension, managed conservatively with good recovery [3]. Bierle et al. reported duodenal ulceration due to ischemia following thoracic endovascular aortic repair, which was successfully treated with endovascular stenting [4]. Meftah et al. presented a case of isolated duodenal necrosis of unclear etiology in a critically ill patient, who unfortunately died intraoperatively [5]. In contrast, a case of duodenal ischemia due to microthrombi in a young patient with paroxysmal nocturnal hemoglobinuria responded well to anticoagulation and eculizumab [6]. These cases highlight variable etiologies, from systemic hypoperfusion to macrovascular and microvascular causes, with mixed outcomes depending on the underlying pathology and the timelines of intervention.

Duodenal ischemia has also been reported secondary to occlusive disease, abdominal aortic aneurysms, complications of percutaneous endoscopic gastrostomy tube placement, endoscopic retrograde cholangiopancreatography, and transarterial chemoembolization [7]. However, in our case, the patient’s operative/postoperative course was complicated by hypotension leading to duodenal mucosal ischemia from hypoperfusion. Unlike most reports involving older patients, our patient was young but had severe microvascular disease due to longstanding type 1 diabetes and end-stage renal disease, amplifying vulnerability to ischemia even without large-vessel occlusion. The cascade of acute coronary syndrome, cardiogenic shock, and escalating vasopressor support mirrors NOMI mechanisms more than thrombotic or embolic ones. Intraoperative findings revealed a well-defined ischemic boundary at the DJ flexure, uncommon in diffuse mucosal cases described in the literature [3-5]. In contrast to conservative-only approaches, we proceeded to diagnostic laparotomy and feeding jejunostomy after multidisciplinary deliberation, recognizing the high risk of perforation and ongoing malnutrition in prolonged ventilator-dependency. Conservative management with fluid resuscitation, bowel rest, antibiotics, and anticoagulation can be tried in non-transmural ischemia. However, surgical management is indicated if there is infarction or perforation. Prognosis of duodenal ischemia varies depending on the underlying cause, speed of diagnosis and severity of injury. Early reversible ischemia has a favorable outcome, while delayed recognition with infarction is associated with high mortality [1].

## Conclusions

In conclusion, this case provides a distinctive addition to the sparse literature on postoperative duodenal ischemia, particularly in the setting of complex comorbidities and hemodynamic collapse. Clinicians should be aware of the possibility of duodenal ischemia in patients with postoperative shock and gastrointestinal bleeding. It emphasizes the spectrum of manifestations from mucosal ulceration to transmural injury and highlights the necessity for prompt diagnosis, tailored intervention, and multidisciplinary collaboration.

## References

[1] Oldenburg WA, Lau LL, Rodenberg TJ, Edmonds HJ, Burger CD (2004). Acute mesenteric ischemia: a clinical review. Arch Intern Med.

[2] Bala M, Kashuk J, Moore EE (2017). Acute mesenteric ischemia: guidelines of the World Society of Emergency Surgery. World J Emerg Surg.

[3] Gupta AK, Lavin A, Kucharik MP, Moseson J (2020). Upper gastrointestinal bleed secondary to duodenal ischemia. Cureus.

[4] Bierle LA, Sweet JM, Chitnavis V (2020). Ischemic duodenal ulceration after thoracic endovascular aortic repair. ACG Case Rep J.

[5] Meftah E, Mohammadzadeh N, Salahshour F (2021). Isolated duodenal ischemia of unknown etiology: a case report. BMC Surg.

[6] Zawawi S, Diamond S, Sharma V (2024). Duodenal ischemia from paroxysmal nocturnal hematuria. Am J Gastroenterol.

[7] Roehlen N, Knoop RF, Laubner K, Seufert J, Schwacha H, Thimme R, Fischer A (2018). Ischemic duodenal ulceration after transarterial chemoembolization for hepatocellular carcinoma: a case report. Case Rep Gastroenterol.

